# A rare case of intracardiac fibrosarcoma with myxoid features inducing venous occlusion in a dog – a case report

**DOI:** 10.1186/s12917-018-1735-2

**Published:** 2018-12-14

**Authors:** Radu Andrei Baisan, Vasile Vulpe, Mircea Lazăr, Sorin Aurelian Pașca

**Affiliations:** 1 Department of Clinics, University of Agricultural Sciences and Veterinary Medicine “Ion Ionescu de la Brad”, Aleea M. Sadoveanu no. 8, 700489 Iași, Romania; 2 Department of Pathology, University of Agricultural Sciences and Veterinary Medicine “Ion Ionescu de la Brad”, Iași, Romania

**Keywords:** Sarcoma, Dog, Metastasis, Right sided heart failure

## Abstract

**Background:**

In both humans and animals, cardiac fibrosarcoma is rare among primary cardiac malignant neoplasia. The overall prevalence of cardiac neoplasia in dogs is low, reported to be between 0.17% and 0.19% of hospital admissions. The aim of this report is to describe the clinical and pathological findings of a dog presenting signs of right sided congestive heart failure due to an intracardiac and venous obstructing mass, diagnosed by histopathology as cardiac fibrosarcoma with myxoid features.

**Case presentation:**

A 7 years old male mix breed Husky weighing 23 kg was presented to our Veterinary Teaching Hospital the owner reporting weight loss, inappetence and exercise intolerance and on presentation exhibited breathlessness and an enlarged abdomen. A 5 minutes six leads electrocardiogram and cardiac ultrasonography were performed using standard, established techniques. Complete blood count, serum liver enzyme activities and renal parameters were assessed. Shortly after the cardiologic examination, the dog died and necropsy examination of the cardiovascular system revealed an elongated and branched mass attached dorsally to the endocardial insertion of the septal tricuspid valve leaflet. This mass extended retrogradely into the lumen of the cervical veins, obstructing the venous flow. Histological diagnosis of the mass was cardiac fibrosarcoma with myxoid features. Multiple metastases were found inside the lungs only.

**Conclusion:**

This is the first report describing a right cardiac fibrosarcoma with myxoid features and venous obstruction in a dog. Cardiac fibrosarcoma is a rare finding, however should be considered when an intracardiac mass is diagnosed.

## Background

Fibrosarcoma is a rare, highly malignant tumour of mesenchymal cell origin. It derives from pathologically transformed spindle shaped fibroblasts with an excessively high division rate [1]. In both humans and animals, cardiac fibrosarcoma is rare among primary cardiac malignant neoplasia [2]. In dogs, approximately 1% of primary cardiac tumours are fibrosarcoma [3]. Fibrosarcoma with myxoid features had been described in human medicine as mxofibrosarcoma, however, veterinary literature describes this type of tumour as either fibrosarcoma or myxosarcoma. According to Milovancev et al. 2015, fibrosarcoma in dog resembles high grade myxofibrosarcoma, while myxosarcoma resembles low grade myxofibrosarcoma [4].

In dogs, the overall prevalence of cardiac neoplasia is low [5], reported to be 0.17% of hospital admissions [6]. Another retrospective large population study reported a prevalence of 0.19%, amounting to 1383 dogs with cardiac tumours out of a total population of 729,265 dogs [3]. Primary cardiac tumours in dogs include chemodectoma, chondrosarcoma, fibrosarcoma, hemangiosarcoma, leiomyosarcoma, lipofibroma, mesothelioma, myxofibroma, myxoma, rhabdomyosarcoma and ectopic thyroid sarcomas [7].

The aim of this report is to describe the clinical and pathological findings of a dog presenting signs of right sided congestive heart failure due to an intracardiac and venous obstructing mass, diagnosed by histopathology as cardiac fibrosarcoma with myxoid features. To author’s knowledge, right sided cardiac fibrosarcoma with myxoid features extending into the venous system had not been reported in veterinary medicine to date. This case-report was structured according to proposed guidelines in the literature [8].

## Case presentation

A 7 years old male mix breed Husky weighing 23 kg was presented to our Veterinary Teaching Hospital the owner reporting weight loss, inappetence and exercise intolerance and on presentation exhibited breathlessness and an enlarged abdomen. Physical examination revealed cyanotic mucosal membranes, severe subcutaneous edema in the head area, thorax and limbs and respiratory effort with a rate of 42 breaths per minute. Palpation of the abdomen revealed a positive ballottement reaction suggesting the presence of ascites. Cardiac sounds were muffled during auscultation and the femoral pulse was fast and weak. A 5 minutes six leads electrocardiogram (PolySpectrum ECG) and echocardiography (Esaote AU5), were performed using previously described methods [9, 10]. Complete blood count, serum liver enzyme activities and renal parameters were assessed.

Electrocardiography revealed a fast sinus rhythm of 140 bpm, absence of respiratory arrhythmia and low voltage QRS complexes (R wave in lead II = 0.09 mV), with a positive polarity in leads I, II, aVL, aVF and negative in leads III and aVR, with a left axis deviation.

**Fig. 1:**
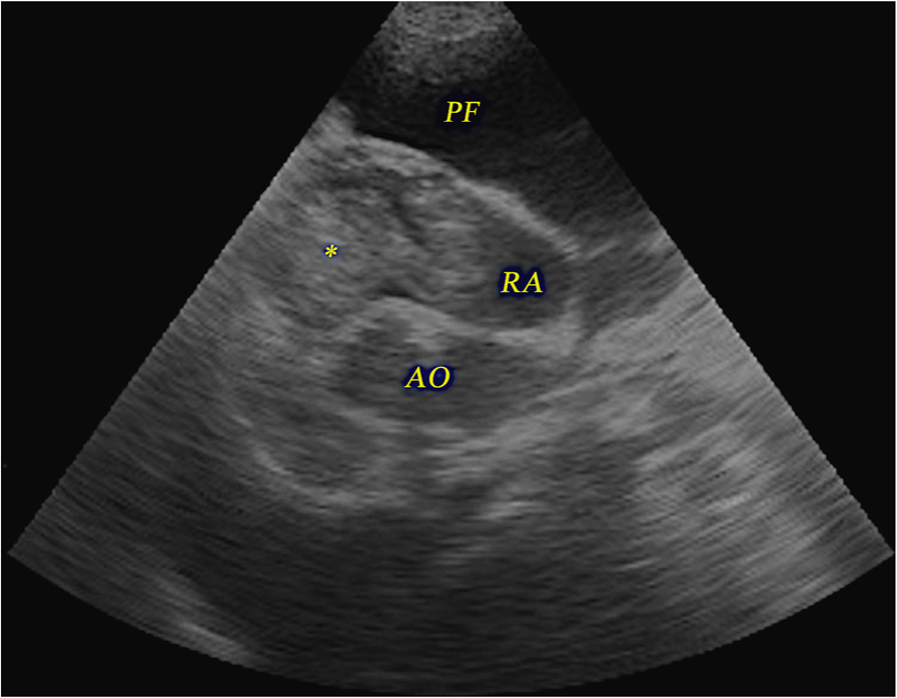
Echocardiography of a dog with intracardiac mass: right parasternal long axis 5 chamber view – a hyperechoic mass (asterisk) is visible inside the right atrial cavity (RA). An anechoic area is visible around the heart represented by pleural fluid (PF); AO – aorta

A brief cardiac echocardiography revealed right atrial and ventricle enlargement with a hyperechoic heterogenous mobile mass of 4.26 × 2.64 cm inside the right ventricle extending into the right atrial cavity through the tricuspid annulus (Fig. 1). A subjective assessment of the left ventricle revealed thickened left ventricular septum and free wall and reduced lumen size, suggesting concentric hypertrophy. The left atrial cavity appeared normal. Also, free pleural fluid was observed. A complete echocardiographic examination was not possible because of the dog’s clinical status. Red and white cell numbers were within the reference range and the haematocrit was mildly decreased Ht%: 38.4 (reference range 40–60%). Serum biochemistry revealed increased activity of serum alanin aminotransferase: 111 U/L (reference ranges 18–86 U/L), alkaline phosphatase: 203 U/L (reference range 12–121 U/L), normal total protein: 5.5 g/dL (reference range 5.4–7.5 g/dL) and increased BUN: 96 mg/dL (reference range 8–29 mg/dL).

Shortly after the cardiologic examination, the dog died and necropsy was performed. The necropsy revealed typical changes of right sided congestive heart failure. Severe cyanosis of oral mucosae, of the tongue and skin were observed. Also, generalized edema of the subcutaneous tissue, more evident in the head area, ventral part of the body and limbs was observed. In both peritoneal and pleural cavities, a large amount (2.5 respectively 0.5 l) of free serous fluid was present. The lung had a pale appearance and higher density than normal at palpation, while the floating thest was negative, likely as a result of compression by pleural fluid.

**Fig. 2:**
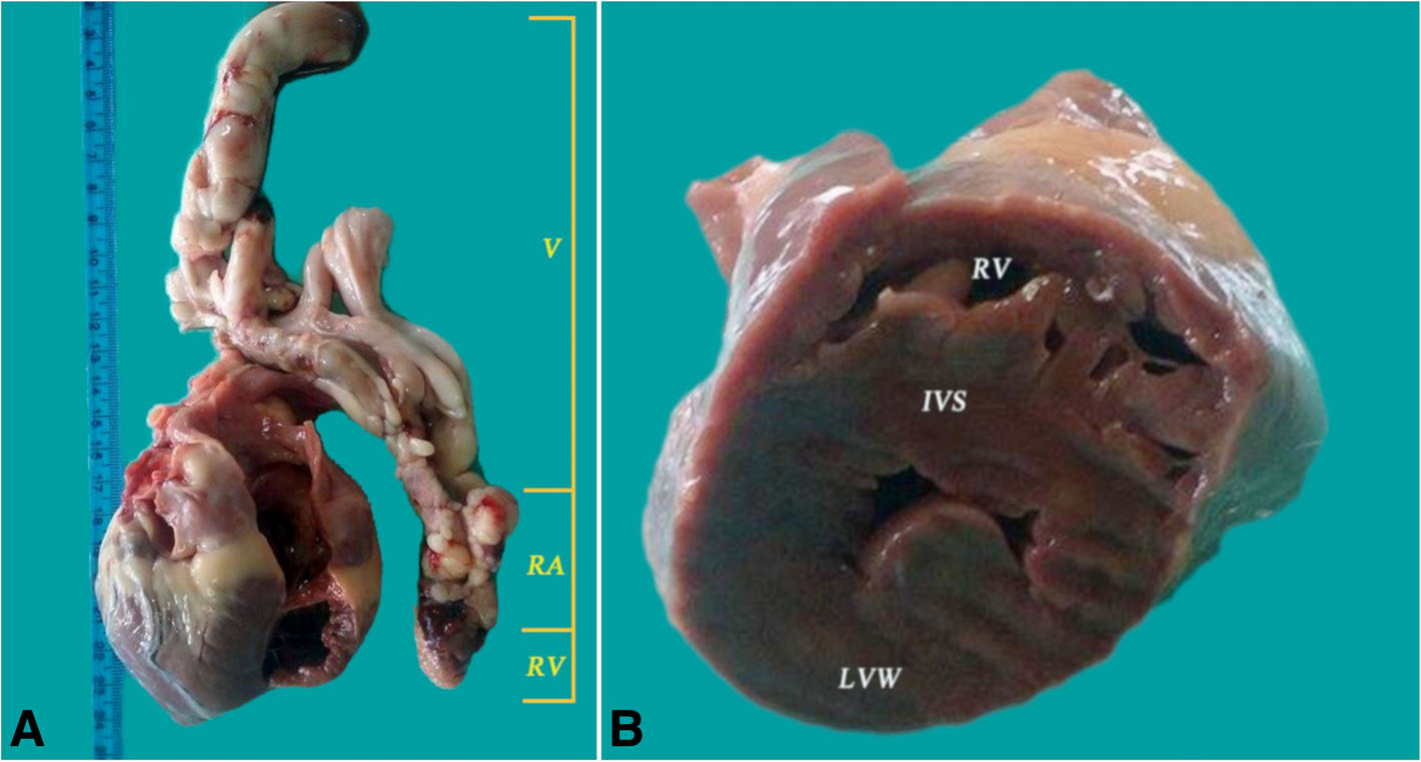
**a** Gross pathology of the heart of a 7 years old male mix-breed Husky. The right heart is dissected and the intracardiac and intravascular mass is presented after the extraction from the heart and vein system showing the localization of the mass: RV – right ventricular mass, RA – right atrial mass, V – intravenous mass; **b** Gross pathology of the heart - section through the papillary muscles, demonstrating a severe concentric hypertrophy with decreased left ventricular cavity; LVW – left ventricular wall, IVS – interventricular septum, RV – right ventricle

Inside the right heart (atrium and ventricle), an elongated and branched mass with a smooth surface was observed. It extended from the right ventricle, retrograde to the right atrium, and then into the cranial vena cava, continued into the right brachiocephalic vein and split into the subclavian and external jugular vein. The subclavian branch was short. From the jugular vein, the tumour extended to the linguofacial and maxillary veins (Fig. 2a). The caudal vena cava and left brachiocephalic veins were enlarged, but no mass was found inside the lumen.

The mass was attached dorsally to the endocardial insertion of the septal tricuspid valve leaflet, suggesting a diagnosis of a primary endocardial tumour. The consistency was firm to elastic, with a smooth surface and an overall whitish colour with small red areas evident dispersed throughout the mass. The sectioned surface of this mass was nonhomogeneous, slightly fatty, with red and yellow striae inside.

The gross examination of the left heart revealed an apparently hypertrophied left ventricle with a reduced ventricular lumen (Fig. 2b). The mitral and aortic valves were normal, as well as the left ventricle outflow tract.

The liver, spleen and pancreas were increased in volume based on gross pathology inspection and a very dark red colour, presumably as a consequence of chronic venous congestion.

During necropsy, multiple fragments were harvested from the intracardiac and intravascular locations of the mass as well as the connection of the mass and the endocardial insertion. Samples were also taken from liver, lung, pancreas, left and right kidney, spleen, brain, both of adrenal glands and myocardium.

Microscopic examination was performed on formalin-fixed and paraffin embedded tissues. The slides were routinely processed and stained with Masson’s trichrome and alcian blue. Selected sections of the tumour were immunostained using the labelled CD34 (QBEnd-10; Dako), vimentin (Novocastra – Liquid mouse monoclonal antibody), desmin (Novocastra – Liquid mouse monoclonal antibody), CD31 (Novocastra – Lyophilized mouse monoclonal antibody), alpha – smooth muscle actin (Novocastra - Lyophilized mouse monoclonal antibody), epithelial membrane antigen (Novocastra - Liquid mouse monoclonal antibody), S-100 protein (Novocastra – Liquid mouse polyclonal antibody) and MUC1 (Novocastra – Lyophilized mouse monoclonal antibody).

**Fig. 3:**
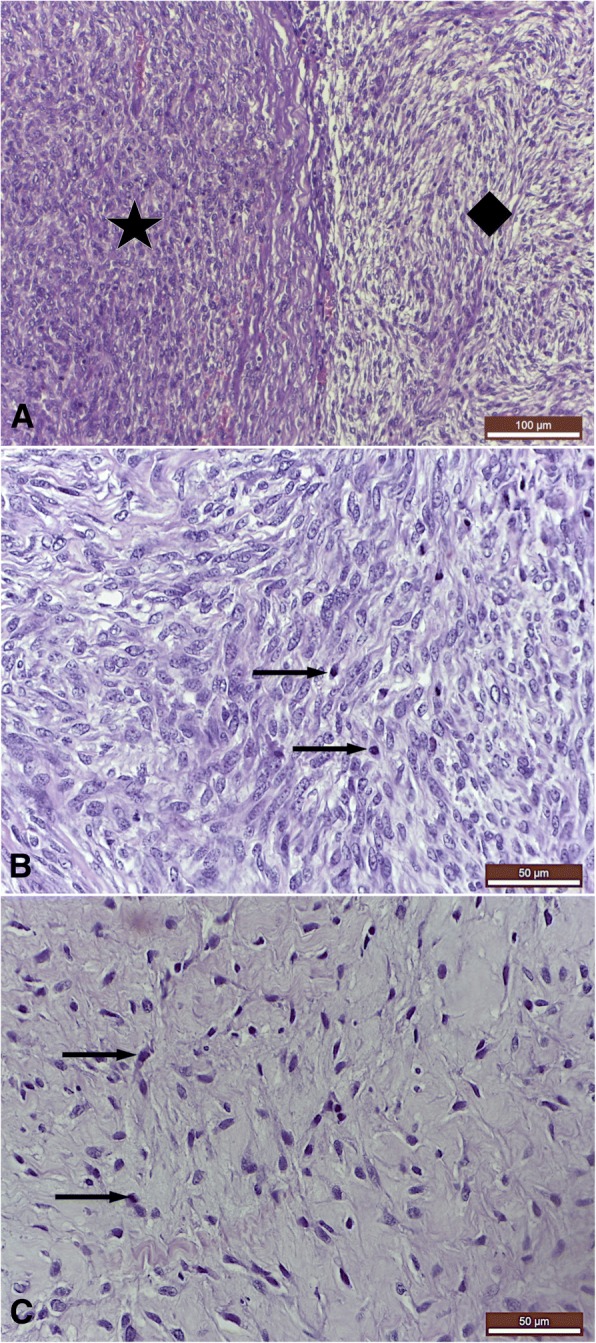
**a** Histolgic aspects of the mixed intracardiac tumor. Two microscopical aspects are visible: compact structure with spindle shape cells (star) consistent with fibrosarcoma and a myxomatous aspect, formed by pleiomorphic cells with cords, trabeculae and small groups distribution (diamond). Fibrosarcoma with myxomatous features - primary tumour; **b** Histological aspect of the fibrosarcomatous area of the mass: multiple mitotic figures are visible on 400× field (arrow); **c** Histological aspect of the myxomatous area of the mass: few mitotic figures are visible on 400× field (arrow). Trichromic Masson stain

Histological examination of the mass revealed a mixed tumour with two microscopical aspects: fibrosarcoma and myxoma. The two different types of the tumor were intercalated, resulting into a tumoral tissue organized in two structures with different architectures (Fig. 3a).

One type was organized in compact structure with spindle shaped cells. These cells had big, irregular and vesicular nuclei (anisokaryosis) with chromatin of a dusty appearance and a marked nucleolus, morphology corresponding to fibrosarcoma tumour. This type showed 8–12 mitotic figures per 400× field consistent with high grade of malignancy (Fig. 3b). The other type had an organisation typical of a myxomatous tumour, formed by pleomorphic cells arranged in cords, trabeculae and small groups. The cells presented spindle, triangular and stellate shapes, anisokaryosis, low mitotic figures (1–3 per 400× field) and syncitia (Fig. 3c). Within the tumor, neoformation blood vessels and a very poor stroma were present. The myxoid matrix was strongly positive for alcian blue stain.

**Fig. 4:**
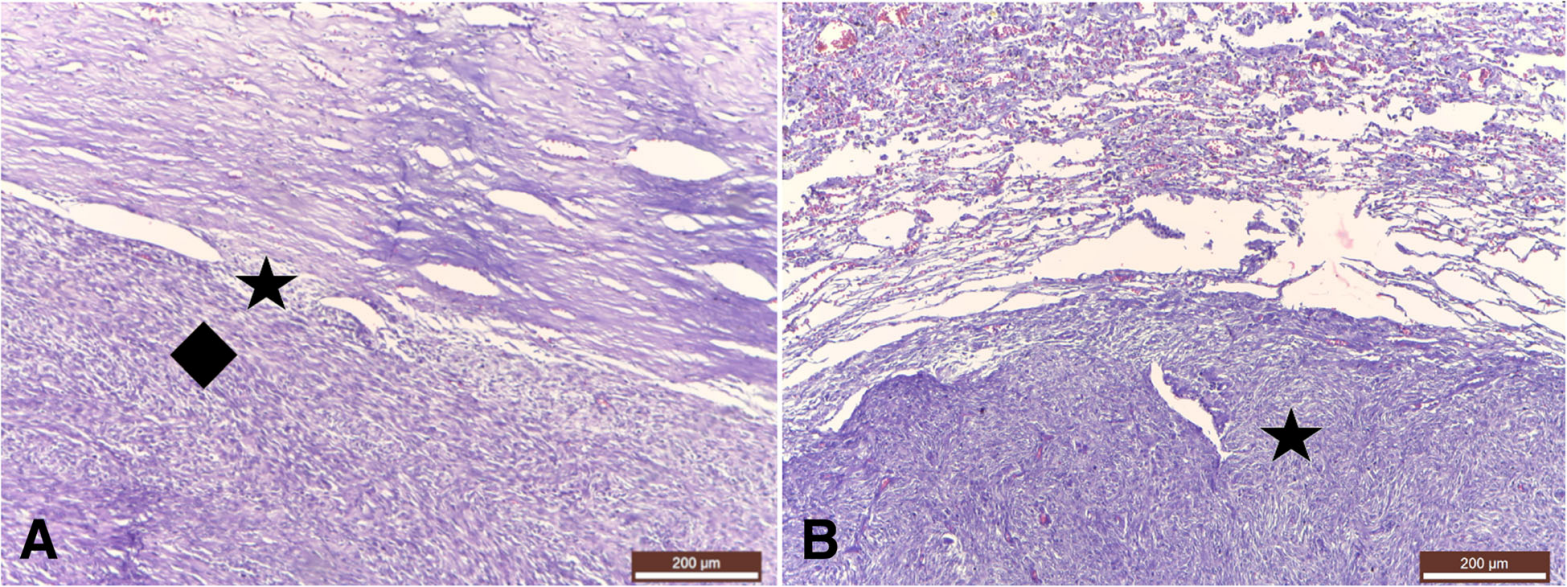
**a** Histologic aspect of the tumoral insertion origin on the myocardial wall. Compact tumoral tissue: fibrosarcoma (diamond) attached to the atrial septal wall (star); **b** Histologic aspect of the lung metastases. Massive tumoral mass (star) and atelectasis by compression around the tumoral metastases. Trichromic Masson stain

The examination of the contact area between the tumor and the atrial wall revealed tumoral cells among the myocardiocytes (Fig. 4a). Within the tumoral mass, extended areas of necrosis were observed, with the persistance of the tumoral arhitecture.

The microscopical lung metastasis had the same mixed structure as the primary tumor. A capsule-like structure represented by condensed lung tissue around the tumoral metastasis, lung atelectasis and compensatory emphysema near the metastasis were observed (Fig. 4b).

**Fig. 5:**
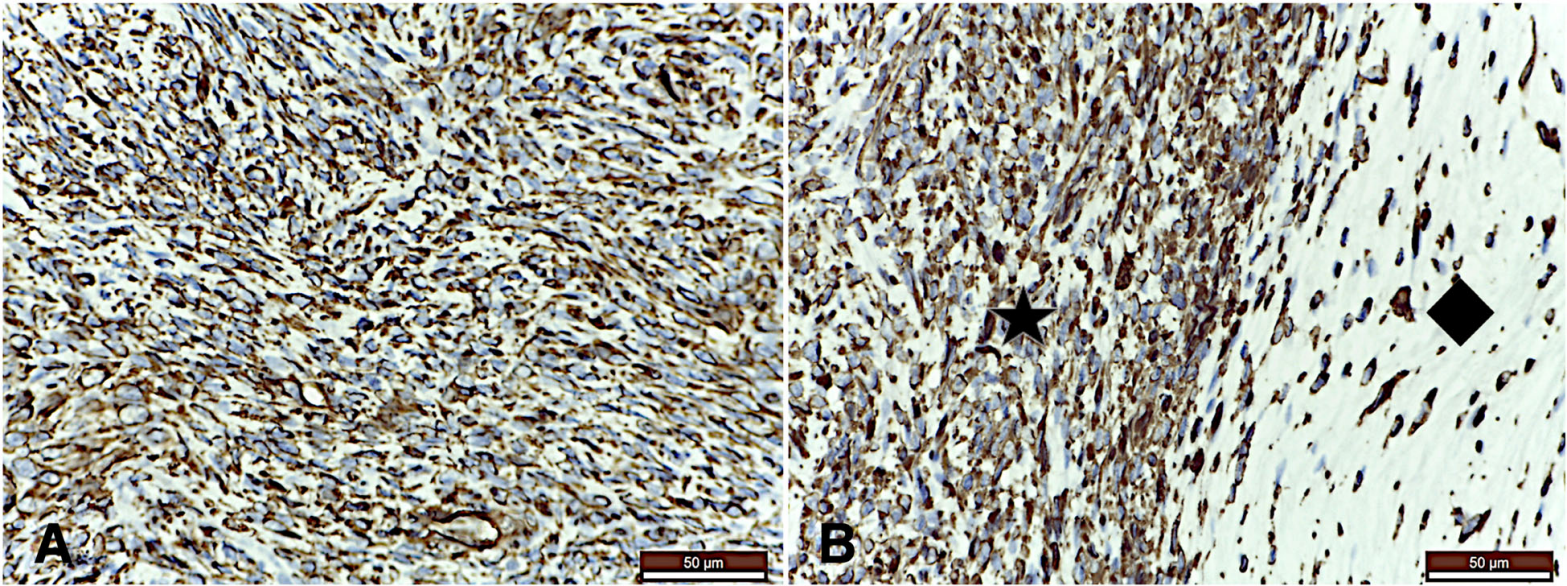
**a** Immunohistochemical stain by vimentin of the tumour. Strong positive stain for vimentin of the fibrosarcomatous part of the mass; **b** two types of architecture are visible within the mass: on the left side of the field, condensed cellularity is visible (star) and on the right side of the field the cells are sparse (diamond) – the cytoplasm of the neoplastic cells stains for vimentin in both structures. Vimentin stain

The fibrosarcomatous type of the cardiac tumor was positive for vimentin (Fig. 5a), S-100 protein and alpha-smooth muscle actin and negative for desmin, CD31, CD34, MUC1 and epitelial membrane antigen. The myxomatous type of the mass was positive for vimentin also (Fig. 5b), alpha-smooth muscle actin, weak positive for S-100 protein and negative for desmin, CD31, CD34, epitelial membrane antigen and MUC1. Desmin was positive only for myocardial tissue where the tumoral mass inserted.

No metastases were found in other organs except the lungs. However, lesions secondary to right sided congestion heart failure were observed in different organs. These lesions consisted of congestion and hypoxia-induced degeneration in the liver, spleen, pancreas and kidneys. Liver congestion was characterised by enlarged sinusoid capillaries, filled with agglutinated red blood cells and within the hepatic lobules, due to hypoxia, large groups of degenerated hepatocytes with pyknotic nuclei were observed. The kidney examination revealed distension of the interstitial capillaries, filled with red blood cells and proximal tubules epitelium degeneration secondary to hypoxia. Histopathological examination of the left ventricle wall revealed an increase in myocardiocytes size and a moderate congestion of the interstitial blood vessels.

## Discussions and conclusions

This paper presents a very rare finding of a right sided congestive heart failure due to a massive cardiac fibrosarcoma with myxoid features associated with pulmonary metastasis in an adult dog. This is the first report of an intracardiac and intravenous obstructive fibrosarcoma with myxoid features in dog.

Several studies in veterinary medicine have reported intracardiac fibrosarcoma in dogs. One study reported a spherical mass of 0.5 cm in diameter in the right atrial surface of the atrial septum [11] while in another case, the tumour was well demarcated involving the subepicardial myocardium of the left ventricle [12]. One report described a large mass at the heart base in a Labrador filling approximately 80% of the left atrial lumen [13]. One paper described a primary malignant mixed mesenchymal tumour of the heart including fibrosarcoma, rhabdomyosarcoma, liposarcoma and chondrosarcoma [14]. Cardiac myxosarcoma in dogs are also very rare. One paper reported an extracardiac intrapericardial myxosarcoma of approximately 3 × 4.5 cm obstructing the right ventricular outflow tract [15].

The dog in this report was presented with severe signs of right sided cardiac heart failure, including peripheral edema and pleural and peritoneal free fluid due to the obstruction of the venous return flow. Electrocardiography revealed moderate sinus tachycardia with marked low voltage of the R-wave and left axis deviation. Tachycardia and low voltage of the R-wave are consistent with pleural and peritoneal effusion [16]. The left axis deviation may have been induced by the left ventricular hypertrophy. This condition have been commonly reported in cats with hypertrophic cardiomyopathy [16]. The increase in circulating liver enzyme activities was probably a consequence of the hepatic congestion secondary to the occlusion of the right ventricular inflow tract, in the absence of other histopathological changes [17]. Echocardiography revealed a hyperechoic mobile mass inside the right ventricle, continued inside the right atrium, consistent with an intracardiac tumour and obliterating the returning venous flow. Necropsy revealed that the mass inside the right ventricle extended into the right atrium and the venous system of the right side of the cranial mediastinum and neck, measuring a total length of 25 cm. The mass probably extended into the right-sided venous system because of the alignment of the right brachiocephalic vein with cranial vena cava compared to the left one which detaches in an angle as it crosses the median plane towards the left side [18].

Histological examination revealed tumour tissue connections with the right atrial septal wall, dorsal to the insertion of the sepal tricuspid leaflet, proving that this mass developed from the heart in the first place and grew retrogradely into the right atrium and venous lumen. The morphological analysis of different type of cells from the mixed tissue mass, the immunohistochemical analysis and the different grades on malignancy based on the mitotic index, as well as the lung metastasis led to the diagnosis of a rare tumor: fibosarcoma with myxoid features originating from the right atrial endocardial wall. Metastases of the same tissue were found only inside the lungs. This finding might be explained by the anatomical path of the blood stream from the right ventricle, on which tumour cells were carried into the lungs.

Echocardiography and gross examination of the heart at post mortem revealed apparent left ventricular hypertrophy in the absence of subaortic stenosis or any changes in the left ventricular outflow tract. These changes may be explained by the low preload of the left ventricle due to the presence of a small amount of blood in the pulmonary circulation. This mechanism has been also associated with cardiac tamponade and acute hypovolemia [19, 20]. In literature, this phenomenon is called pseudohypertrophy due to its transitory nature once blood reperfusion is achieved [21].

Fibrosarcoma with myxoid features, although a very rare finding, should be included in the differential diagnosis of intracardiac masses that may obliterate the blood flow and develop metastases in other organs.

## Abbreviations

BUN: Blood urea nitrogen
Ht%: Haematocrit
